# Efficacy of ^177^Lu-Dotatate Induction and Maintenance Therapy of Various Types of Neuroendocrine Tumors: A Phase II Registry Study

**DOI:** 10.3390/curroncol28010015

**Published:** 2020-12-21

**Authors:** Golmehr Sistani, Duncan E. K. Sutherland, Amol Mujoomdar, Daniele P. Wiseman, Alireza Khatami, Elena Tsvetkova, Robert H. Reid, David T. Laidley

**Affiliations:** 1 Department of Nuclear Medicine, Department of Medical Imaging, Western University, London, ON N6A 3K7, Canada; duncan.sutherland@lhsc.on.ca (D.E.K.S.); alireza.khatami@lhsc.on.ca (A.K.); roberth.reid@lhsc.on.ca (R.H.R.); david.laidley@lhsc.on.ca (D.T.L.); 2 Department of Radiology, Department of Medical Imaging, Western University, London, ON N6A 3K7, Canada; amol.mujoomdar@lhsc.on.ca (A.M.); daniele.wiseman@lhsc.on.ca (D.P.W.); 3 Department of Medical Oncology, Western University, London, ON N6A 3K7, Canada; elena.tsvetkova@lhsc.on.ca

**Keywords:** neuroendocrine tumor, NET, PRRT, peptide receptor radionuclide therapy, DOTATAE, Lu-177

## Abstract

Peptide receptor radionuclide therapy (PRRT) has been recently established as a treatment option for progressive gastro-entero-pancreatic neuroendocrine tumors (NETs) including four 200 mCi induction cycles. The purpose of this phase 2 trial is to expand use of PRRT to different types of NETs with the application of dose adjustment and evaluate value of maintenance therapy in patients who had disease control on induction therapy. Forty-seven PRRT naïve NET patients with different primary origin received ^177^Lu-DOTATATE induction therapy, ranging from 75 to 150 mCi per cycle, based on patients’ clinical status such as liver and renal function, extent of metastases, and previous therapies. Thirty-four patients underwent additional maintenance therapy (50–100 mCi per cycle) following induction course until they developed disease progression. The estimated median progression-free survival (PFS) was 36.1 months. The median PFS in our MNET subgroup was 47.7 months, which is markedly longer than NETTER-1 trial with median PFS of 28.4 months. The median PFS was significantly longer in patients who received PRRT as first-line treatment after disease progression on somatostatin analogs compared to patients who received other therapies first (*p*-value = 0.04). The total disease response rate (DRR) and disease control rate (DCR) was 32% and 85% based on RECIST 1.1 and 45% and 83% based on Choi criteria. This trial demonstrates longer PFS with the addition of low dose maintenance therapy to induction therapy compared to NETTER-1 trial that only included induction therapy. Also, we observed considerable efficacy of PRRT in various types of advanced NETs.

## 1. Introduction

Neuroendocrine tumors (NETs) represent heterogeneous group of malignancies that can involve almost any organ in body [1]. The incidence and prevalence of NETs are increasing due to advancements in medical imaging and improved medical awareness about these types of tumors and their symptoms [1,2,3]. The 5- and 10-year overall survival (OS) rates in NET patients based on recent SEER data are 39.4% and 18.1%, respectively [4]. NETs are classified as functional and non-functional based on the presence or absence of hormonal hypersecretion. Patients with non-functional NETs lack typical hormonal symptoms and tend to present with more advanced disease [5,6]. Nearly 60–80% of those with non-functional diseases are metastatic at diagnosis [5]. The gastrointestinal tract and lungs are the most common origin sites of NETs [7], and the liver is the most common metastatic site [8]. The treatment of NETs includes surgery with curative intent [9,10], somatostatin analogs (SSA) for residual disease, local therapies such as chemo-embolization [11], radio-embolization [12] and radiofrequency ablation [13,14] for hepatic metastases, and systemic therapies such as capecitabine-temozolomide chemotherapy [15] or m-TOR inhibitor such as Everolimus [16] for advanced metastatic cases.

The majority of these NETs express a high number of somatostatin receptors (SSTRs) on their cell membranes. Stimulation of SSTRs has antisecretory and antiproliferative effects, and therefore, SSAs, such as octreotide and Lanreotide, have been used for control of tumor growth and symptoms [17,18,19]. Peptide receptor radionuclide therapy (PRRT) using radiolabeled SSAs has recently shown more considerable promise for the treatment of advanced, metastatic well-differentiated NETs [5,6,20,21,22,23]. The most common radionuclide for this purpose is Lutetium-177 (^177^Lu), which is a beta emitter and labeled with SSAs in the form of ^177^Lu-DOTA^0^-Tyr^3^-Octreotate (^177^Lu-DOTATATE) [24]. The beta particle of ^177^Lu has a maximum energy of 0.49 MeV, a maximum range of 2 mm, and a half-life of 6.7 days [25].

Recently, the NETTER-1 phase 3 trial showed superior efficacy of PRRT compared to octreotide long-acting repeatable (LAR) in midgut NETs (MNETs) [21]. This trial was instrumental in the Food and Drug Administration (FDA) approval of ^177^Lu-DOTATATE in 2018 for the treatment of unresectable, well- or -moderately-differentiated gastro-entero-pancreatic (GEP) NETs. However, the trial scope was limited to induction course of treatment, including 4 cycles of therapy, only MNETs were included, and all patients were given a fixed dose of PRRT. Maintenance therapy may slow down the growth of advanced cancer and potentially can lengthen a patient’s life. Additionally, giving a fixed dose of such therapy to all patients regardless of each case’s clinical status may underexpose or overexpose patients to the radiation. Given the limitation of the NETTER-1 trial, the purpose of this phase 2 trial is to evaluate the expanded use of PRRT to multiple different types of NETs with the application of dose adjustment and evaluate the value of maintenance therapy in patients who had disease control on induction therapy.

## 2. Experimental Section

Ongoing single-center, single-arm, open-label phase 2 registry study started in our center in July 2014. It was approved by the research ethics board (Ethics no:104378). All participants provided written consent.

Although this trial has been open to patients with the previous PRRT, only PRRT naïve cases were included in this analysis. The eligibility criteria included the presence of a positive tumor on SSTR scan (Krenning score 3 and 4) within 16 weeks of enrollment, a Ki-67 < 20%, a life expectancy > 26 weeks from enrollment, serum creatinine ≤ 130 μmol/L or GFR > 50 mL/min/1.73 m^2^, hemoglobulin ≥ 90 g/L, white blood cell ≥ 3 × 10^9^/L and platelets ≥ 100 × 10^9^/L within 4 weeks of enrollment, liver function tests ≤ 3 times of the normal limits, ECOG (Eastern Cooperative Oncology Group) Performance Scale Score ≤ 2 measured within 4 weeks of enrollment. The exclusion criteria included pregnancy, breastfeeding, the potential for curative surgery, prior radiation therapy to more than 25% of the bone marrow; patients should not have surgery, radiation therapy, radioisotope therapy, change in Sandostatin LAR dosage, cytotoxic chemotherapy, embolization or other investigative treatments (interferons, mTOR inhibitors) within 12 weeks of enrollment, known brain metastases unless these metastases have been treated and/or are stable ≥ 24 weeks before enrollment, uncontrolled diabetes mellitus, any significant uncontrolled medical, psychiatric or surgical condition which may interfere with completion or conduct of the study.

Patients retained the right to withdraw from the study at any time. The investigator also had the right to withdraw a subject from the study in the event of intercurrent illness or other reasons concerning the health or well-being of the subject, or an inability to cooperate with the study protocol.

### 2.1. Protocol

The majority of patients were treated in outpatient setting in the Nuclear Medicine Department. Sandostatin LAR was discontinued 7 days before and for 7 days after ^177^Lu-DOTATATE therapy. Short-acting octreotide is discontinued 1 day before and after therapy. Nonsteroidal anti-inflammatory drugs (NSAIDs) also are stopped for 1 week before and after treatment. All herbal medicine must be discontinued. Patients received granisetron 30 min prior to administration of amino acids solution as well as dexamethasone unless they had poorly controlled diabetes or another contraindication. Amino acids infusion (1 L of 2.5% arginine, 2.5% lysine) was started 60 min before the ^177^Lu-DOTATATE injection and continued for 4 h until completion. ^177^Lu-DOTATATE co-infusion took 30 to 45 min. Post therapy ^177^Lu-DOTATATE scan for disease evaluation and monitoring was typically performed at 24 h. Blood workup was done weekly until the next cycle of therapy. Full induction therapy included 4 cycles of therapy 10 weeks (8–12 weeks) apart. Radioactive ^177^Lu-DOTATATE doses (administered activity) were given in the range of 2.78–5.55 GBq (75 to 150 mCi). Any dose could be increased or decreased by 25 mCi (0.92 GBq) with each subsequent therapy to a maximum dose of 150 mCi (5.55 GBq) and a minimum dose of 75 mCi (2.78 mCi). Dose modification depended on the tolerability of previous treatments and multiple factors elaborated in Table 1.

All patients underwent computed tomography (CT) scans within 16 weeks prior to treatment to document the baseline extent of disease, and 16 weeks after termination of fourth cycle to evaluate response to the induction therapy. As opposed to the NETTER-1 trial, when patients had CT scans every 3 months, we used 24 h post therapy ^177^Lu-DOTATATE scan, including the total body and single-photon emission computed tomography-computed tomography (SPECT/CT), to assess for response or progression during the induction therapy.

If the tumor control was achieved after induction therapeutic stage (response or stable disease), patients entered the maintenance stage with an average of 75 mCi (2.78 GBq) of ^177^Lu-DOTATATE (range of 50–100 mCi (1.85–3.70 GBq) depending on patient status) that was administered every 6 months for up to 4 years after induction therapy or until disease progression. For response evaluation, CT scan performed every 4 months after each maintenance phase treatment. Post therapy ^177^Lu-DOTATATE was also used to determine the extent of disease and evaluation of response during the maintenance phase before obtaining the CT.

^177^Lu-DOTATATE and amino acid solutions were provided by IDB Holland, an Advanced Accelerator Applications company. ^177^Lu-DOTATATE was compounded locally. Funding for the isotope and amino acids was provided by Cancer Care Ontario.

The primary objectives of the study were the PFS and OS on ^177^Lu-DOTATATE treatment. The secondary objectives were response rate and the safety of ^177^Lu-DOTATATE. The safety data will be published separately.

The imaging-based response to induction therapy was evaluated by RECIST 1.1 and Choi criteria and reviewed by two interventional and abdominal radiologists with extensive experience reviewing NETs. Choi criteria is typically used for GIST or HCC tumors [26]. The response categories included a complete response (CR), partial response (PR), stable disease (SD), and progressive disease (PD). We used functional imaging as a guide for the selection of target lesions. The final response was also evaluated by a nuclear medicine physician who reviewed functional studies during therapy. The imaging-based response rate was only evaluated for the induction therapy.

### 2.2. Statistics

Descriptive data are presented as mean ± standard deviation (SD) or percentage as appropriate. The median PFS and OS were estimated by the Kaplan–Meier method. The log-rank test was used for comparison of survival between groups. A *p*-value of 0.05 or less was considered statistically significant (95% confidence interval).

## 3. Results

**Figure 1:**
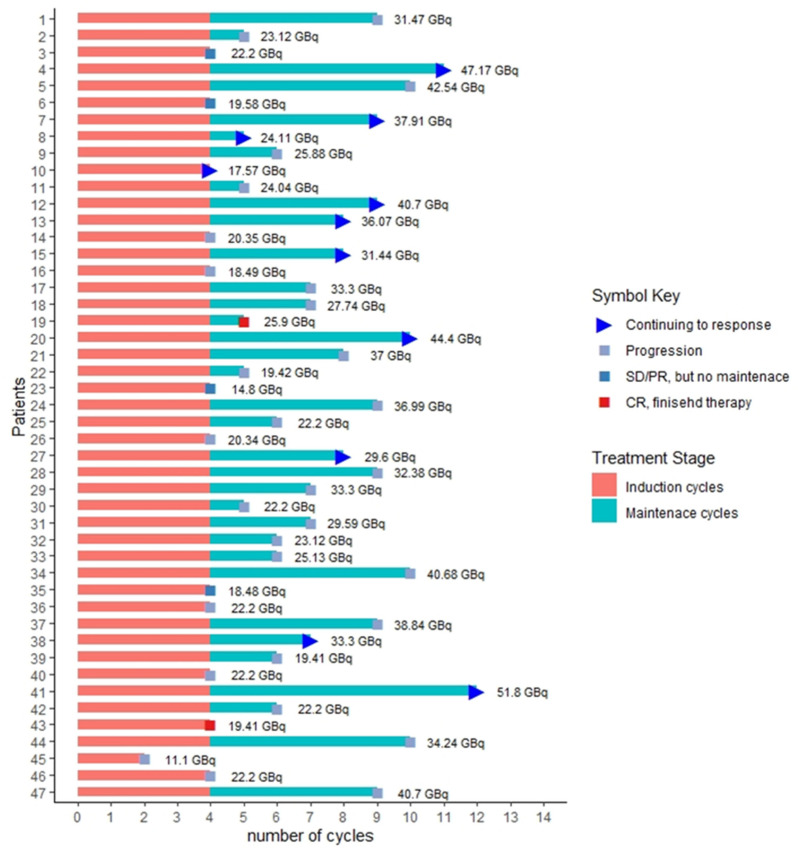
Swimmer plot representing the total number of cycles and cumulative dose for each patient. SD = stable disease. PR = partial response.

The study included a total of 52 PRRT naïve patients with 47 patients included in this analysis. Three patients were withdrawn from the trial due to safety concerns secondary to unreported dementia and cognitive decline. Two patients withdrew consent. Forty-six patients completed induction therapy (four cycles), one patient with lung NET received two cycles, and therapy stopped due to disease progression. Thirty-four patients entered the maintenance therapy phase and were treated until disease progression, or death from other causes or 4 years after induction therapy, whichever came first. Patients received between 1 to 8 cycles of maintenance therapy with a median of 3.5 cycles. Twelve patients did not receive maintenance therapy, including: Six out of 12 patients had progressive disease; one patient with complete response didn’t receive further maintenance therapy; one patient with partial response had GFR reduction to less than 50 mL/min/1.73 m^2^; two patients with partial response withdrew further treatment; one patient with stable disease (based on RECIST 1.1 criteria) had a mixed response with increasing size of hepatic metastases and decreasing size of extrahepatic metastases and was referred for hepatic directed therapy based on tumor board discussion; one patient with reduction is the size of metastases within stable range of RECIST 1.1 criteria (<30% reduction) referred for surgical resection of pancreatic metastases based on tumor board review. The total number of ^177^Lu-DOTATATE therapy (induction + maintenance) was 309 cycles in 47 patients, including 186 induction and 123 maintenance cycles. Figure 1 shows a swimmer plot of all patients depicting course of therapy, total cycles with separation of induction and maintenance cycles and the cumulative dose each patient received. Thirty-three out of 47 patients (70%) received modified doses at some point during the treatment based on clinical status.

The demographic and relevant clinical information is summarized in Table 2.

**Figure 2:**
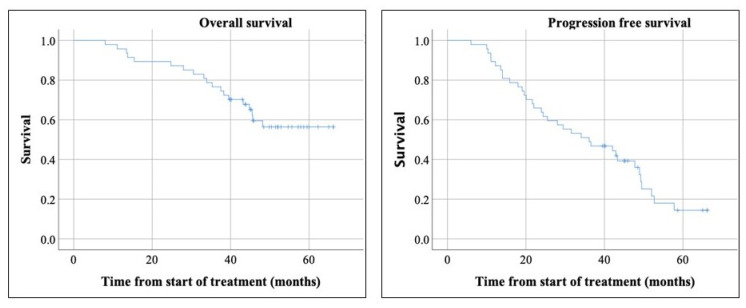
The overall survival did not reach median. The median progression free survival was 36.1 months.

**Figure 3:**
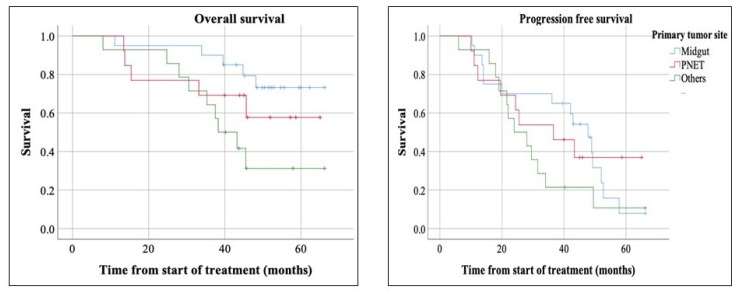
Overall survival (OS) and Progression-free survival (PFS) based on tumor types. The median OS was significantly longer in patients with midgut neuroendocrine tumors (NETs) (*p*-value = 0.039). Estimated median PFS was 47.7 months in midgut NETs, 36.5 in pancreatic NETs, and 23.9 months in other types of NETs, which the difference was not statistically significant (*p*-value = 0.35).

The median follow-up was 51.9 months with the reverse Kaplan-Meier method. Median PFS was 36.1 months, with a median of 47.7 months in MNETs, 36.5 in pancreatic NETs (PNETs), and 23.9 months in other types of NETs (Figure 2 and Figure 3). These differences were not statistically significant.

The median PFS was longer in grade 1 NETs measuring 47.7 months compared to 29 months in grade 2 NETs. While lower grade tumors trended towards a better outcome and may be expected given the importance of tumoral grading in patient’s prognosis, but this did not reach statistical significance (*p*-value = 0.43).

**Figure 4:**
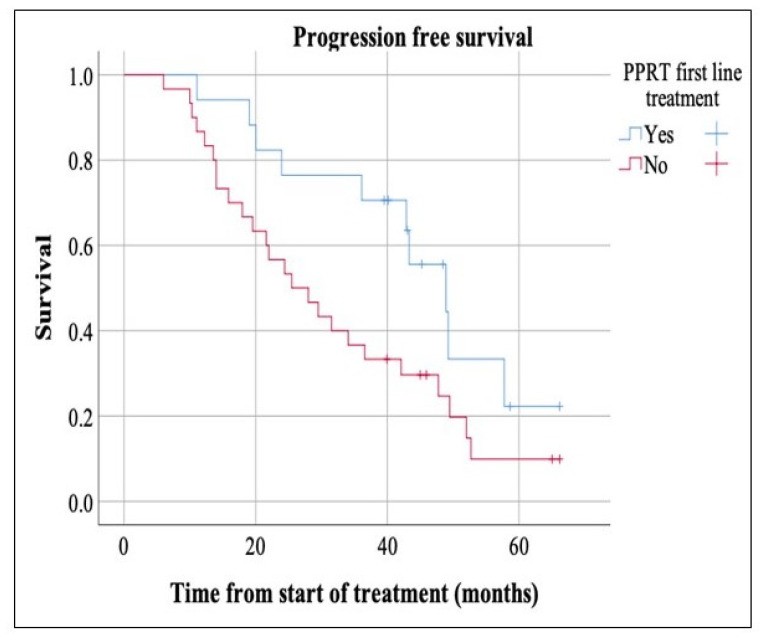
The median PFS was markedly longer in patients who received peptide receptor radionuclide therapy (PRRT) as first therapy post disease progression/metastases, measuring 48.9 months compared to 25.5 months in the other group (*p*-value = 0.04).

Apart from SSAs that all patients had received, there was significant heterogeneity in prior treatments of participants (Table 2). A total of 63.8% (30 patients) received some form of therapy, such as chemotherapy, radiation, chemoembolization and/or RFA before PRRT. The remaining 36.2% (17 patients) were treated with PRRT as an upfront first therapy after disease progression on SSAs. The median PFS was significantly longer in patients who received PRRT as first line treatment after disease progression on SSAs, measuring 48.9 months compared to 25.5 months in the other group (*p*-value = 0.04) (Figure 4).

Median OS for all NETs was not reached (mean of 51.3 months) (Figure 2). Subgroup analysis showed significantly longer OS in MNET compared to PNET and other types of NETs (*p*-value = 0.039). The median OS was not reached for MNET and PNET. The median OS in other types of NETs was 38.3. The median OS was not reached in grade 1 and 2 NETs. A total of 19 patients died during the study window (median follow-up of 51.9 months), with 15 being from disease progression and 4 from other causes.

The response rate of induction therapy based on RECIST 1.1 and Choi criteria is summarized in Table 3. The response rate based on primary tumor site is summarized in Table 4.

Two patients had a CR to PRRT: One of them had primary eustachian tube NET with recurrence post operation. The patient remained progression-free for two years and then was referred to another center for radiation therapy of right vestibular schwannoma. The second patient had renal NET post nephrectomy with metastases to liver and lymph node, with the metastases resolving on post-therapy imaging and CT scans. The patient received one additional maintenance therapy and eventually developed a new metastatic hepatic lesion three years after the last PRRT cycle.

## 4. Discussion

This phase 2 trial analyzed the efficacy of induction and maintenance PRRT in different types of NETs. Forty-six out of 47 patients received complete induction therapy, including 4 cycles; one patient received incomplete induction therapy, including two cycles; and 34 out of 47 patients received additional maintenance therapies with a median of 3.5 cycles. The estimated overall median PFS was 36.1 months, which is comparable to the studies performed on 310 GEP NETs (three to four cycles) with a PFS of 33 months [20]. Similarly, our median PFS of 36.5 months in PNETs is comparable to a study performed on 68 PNETs (four to six cycles) with a PFS of 34 months [27]. Our estimated median PFS of 47.7 in MNETs was longer than similar studies performed on 43 MNET (five cycles) and 61 MNET with median PFS of 36, and 33 months [28,29]. Also, the median PFS in our MNET subgroup is markedly longer than the NETTER-1 trial with median PFS of 28.4 months, which included 4 cycles of induction therapy (7.4 GBq or 200 mCi) in MNETs [30]. This shows improvement in median PFS with the addition of low dose maintenance therapy in patients who have had tumor control post induction therapy, compared to the NETTER-1 trial. As famous 4Rs of radiobiology (repair of DNA damage, redistribution of cells in the cell cycle, repopulation, and reoxygenation of hypoxic tumor areas) supports the idea of fractionated radiation, similar principles can be applied to targeted radionuclide therapy, especially with beta-emitters such as ^177^Lutetium [31]. Maintenance therapy can potentially slow down the growth of cancer cells after the initial induction therapy and improve survival.

Median PFS in MNETs, PNETs and other types of NETs (47.7, 36.5 and 23.9 months, respectively) was markedly longer than alternatives, such as Everolimus with 11 months in RADIANT 3 and 4 trial [16,32], and octreotide LAR with 14.3 months in PROMID study [18], and Sunitinib with 12.6 months [33]. Also, our median PFS in combined MNETs and PNETs was 43.3 months which is longer than Lanreotide with median PFS of 38.5 months in enteropancreatic NETs in CLARINET trial [17]. The estimated median OS was longer in MNETs (*p*-value = 0.039) compared to PNETs and other types of NETs, which is likely related to overall better prognosis of MNETs compared to other types of NETs [4].

Additionally, our study demonstrated that patients who received upfront PRRT following progression or metastatic spread had significantly longer PFS compared to those who received other forms of treatment before PRRT (48.9 months compared to 25.5 months, *p*-value = 0.04). This finding potentially supports the earlier introduction of PRRT in the treatment of metastatic inoperable NET following progression on SSAs. It is undoubtedly hypothesis-generating trial and future studies are warranted. It is noteworthy to mention that most of patients who received PRRT upfront, had MNETs.

The total disease response rate (DRR) post induction therapy, including CR and PR, was 32% (15 patients) based on RECIST 1.1 criteria. Our DRR is similar to reported in Rotterdam study (31% based on Southwest Oncology Group (SWOG) criteria) and higher than it was reported in NETTER-1 trial with DRR of 18% (18 patients) [5,20]. Our total DRR of 32% was higher than DRR of 18% (18 patients) in NETTER-1 trial. However, our MNET subgroup DRR was 5% which was lower than NETTER-1 trial [21]. Our highest DRR was observed in PNETs and lowest in MNETs. This is likely related to the lower radiosensitivity of MNETs owing to lower replication or mitosis rates of intestinal cells [31]. Similar to NETTER-1 trial, this study shows improved objective DRR of PRRT compared to alternative treatments such as octreotide LAR control group of NETTER-1 trial with DRR of 3% (3 patient) [22] and Everolimus with DRR of 5% and 2% observed in RADIANT-3 and -4 trial [16,32] with the added value of including different types of NETs.

The disease control rate (DCR) post PRRT, including a CR, PR, and SD, was 85.1% based on RECIST 1.1 criteria and 83% based on Choi criteria. The difference in disease control rate between Choi and RECIST 1.1 Criteria is small and is likely due to different cut-off values defining PD and PR in RECIST 1.1. and Choi criteria. The solitary use of the tumor size for evaluation of response in RECIST criteria has some pitfalls and limitations. Choi criteria include reduction of tumor attenuation as part of the response to therapy, which reflects necrotic changes of a tumor in response to treatment before a significant reduction in size. Our DCR (85.1%) is comparable to previous studies. For example, Rotterdam study on 310 GEP NETs showed DCR of 81–88% [5] and other studies performed on 68 PNET, 43 MNET, and 61 MNET with DCRs of 85.3%, 84% and 91.8% respectively [27,28,29]. A recently published meta-analysis, including 1127 patients who met RECIST 1.1 criteria, showed DRR of 35% and DCR of 83% [34]. We observed comparable DRR and DCR to the previous studies with the added value of administration of modified administered activity in patients with reduced renal or liver function, extensive bone/marrow metastases, or previous nephrotoxic or marrow-toxic treatments. As external radiation and chemotherapy are customized to patients based on disease burden and comorbidities, PRRT dose also should be tailored to a patient’s condition. Administering a fixed dose to all patients may cause significant toxicity resulting in loss of PRRT advantage in regard to better tolerability to alternatives [35].

**Figure 5:**
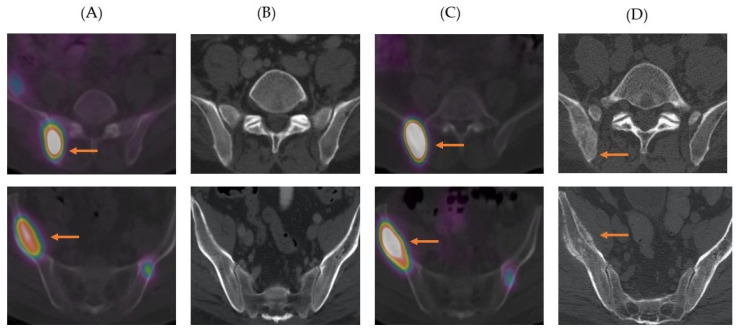
Primary pancreatic neuroendocrine tumor with metastases to liver, lymph nodes and bones. (**A**). Selected axial ^177^Lu-DOTATATE SPECT/CT image post first cycle of therapy shows multi-focal uptake in iliac bones. (**B**). Selected axial CT image before therapy shows no corresponding osseous lesion. (**C**). Selected axial ^177^Lu-DOTATATE SPECT/CT post fourth cycle of therapy shows persistent uptake at the similar locations. (**D**). Selected axial CT image after fourth cycle of therapy demonstrates sclerotic changes in the right iliac bone posteriorly and pathologic fracture and underlying sclerotic changes in the wing of right iliac bone.

In contrast to the NETTER-1 trial, post therapy ^177^Lu-DOTATATE scan was used to monitore disease during induction therapy. Cross-sectional imaging was performed only four months after completion of induction therapy. Besides guidance for the detection of target lesions on CT scan, we observed the added value of better evaluation of osseous metastases. Figure 5 show selected images of a patient with widespread metastases to the liver, lymph nodes, and bones.

The immediate post therapy scan after the first cycle of treatment showed radiotracer avid lesions in bilateral iliac bones, which were not discernible on the CT scan performed 12 days earlier. These avid lesions persisted on post therapy scans obtained after 4 cycles of induction treatment without the development of new lesions. The final CT scan performed after induction therapy showed new sclerotic osseous lesions. Perhaps in the trials that they do not acquire or use post therapy scans, these lesions could have been misinterpreted as new metastases and patient would have been withdrawn from the therapy. The sclerotic lesions may represent remodeling changes post response to the treatment or delayed presentation of lesions on anatomical imaging. So, we firmly believe that functional imaging should be used for response evaluation in conjunction with anatomical imaging. ^177^Lu-DOTATATE affinity for somatostatin receptors is 9-fold higher than ^111^In-pentetreotide (Octreoscan) [36]. Additionally, ^177^Lu-DOTATATE is given in therapeutic quantities in PRRT. These offer a unique opportunity to acquire highly sensitive SPECT/CT studies. Since patients are already receiving a therapeutic dose of ^177^Lu-DOTATATE, the only cost is technologist camera time, which can be saved by omitting cross-sectional imaging for monitoring disease during induction therapy. Nonetheless, follow-up with anatomical imaging has a crucial role in the delineation of lesions and the detection of progressive cases with the dedifferentiation of tumors showing interval reduction in uptake [35].

Our study extends the benefit of PRRT to a broader range of NET types compared to the NETTER-1 trial and previous studies, which were mainly on GEP NETs. Our study included rare NETs such as metastatic renal and recurrent eustachian tube NETs that demonstrated CR to therapy; ampullary NET with PR; metastatic bilateral ovarian NET, rectal NET, malignant pheochromocytoma, and two lung NETs that had SD.

A limitation of our study is small number of non-GEP NETs, which are rare by nature. More studies are required about the assessment of PRRT efficacy on these rare types of NETs. Another limitation of this study is including only grade 1 and 2 NETs (Ki-67 < 20%), that is similar to the NETTER-1 trial. Based on the 2010 WHO criteria, grades 1 and 2 NETs represent well-differentiated disease [37]. However, the definition of grade 3 has changed significantly in the new 2017 WHO criteria. The grade 3 category in the 2010 WHO grading system did not distinguish NETs from NECs. Based on the 2017 WHO criteria, grade 3 with Ki-67 > 20% can be categorized as either well-differentiated or poorly differentiated based on histopathological characterization [38]. So, it appears that well-differentiated versus poorly differentiated is a better inclusion criterion to select NET patients for PRRT rather than Ki-67.

## 5. Conclusions

Our phase 2 trial demonstrates longer PFS with the addition of low dose maintenance therapy to induction therapy compared to the NETTER-1 trial that only included induction therapy. Also, our study indicates considerable efficacy of PRRT in various types of advanced NETs, longer PFS in patients who received PRRT as first-line treatment after disease progression on SSAs, comparable DCR and DRR to previous studies with the added value of dose adjustment based on patient’s clinical status, and benefit of post therapy ^177^Lu-DOTATATE scan application in conjunction with CT scan in the evaluation of response to therapy. Better inclusion criteria such as the proper implication of new 2017 WHO criteria and utilization of new advanced SSTR imaging such as ^68^Ga-DOTATATE PET/CT in patient selection can potentially add the number of patients benefiting from this targeted therapy.

## Figures and Tables

**Table 1:** Dose regimen determination and modification for treatment. * Based on Cockcroft-Gault formula of creatinine clearance.

Risk Factor	Dose				Notes
	150 mCi (5.55 GBq)	125 mCi (4.62 GBq)	100 mCi (3.7 GBq)	75 mCi (2.78 GBq)	
Age (years)	<65	65–75	>75	If more than 2 risk factors identified.	-
GFR *	≥65 corrected	56–65 corrected	50–55 corrected		<50 corrected: Tx not offered.
Liver disease involvement (CT/MRI/^177^Lu scan) and LFT **	≤70% of liver involved approximately		>70% of liver involved		Outside 3 × normal limits: Therapy not offered.
Platelets	≥100 × 10^9^/L	≥100 × 10^9^/L	≥100 × 10^9^/L		<100 × 10^9^/L (<95 × 10^9^/L for subsequent Tx).
WBC	≥3 × 10^9^/L	≥3 × 10^9^/L	≥3 × 10^9^/L		ANC < 1.5 × 10^9^/L: Tx not offered.
Previous nephron- and/or marrow-toxic therapies	None	1 course of chemotherapy or ≤4 RIT with dose < 800 mCi.	>1 course of chemotherapy or >4 RIT with dose > 800 mCi.		
Weight(kg)	≥60	50–59	<50		Dose to be not >2.5 mCi/kg (0.07 GBq/kg)
Bone and marrow disease involvement of axial skeleton	≤5 bone metastases	6–10 bone metastases	>10 bone metastases	Diffuse bone marrow involvement	

** LFT: Liver function tests (Alkaline phosphatase, Aspartate transaminase, Alkaline phosphatase, albumin). * GFR: glomerular filtration rate (mL/min/1.73 m^2^). RIT: radioimmunotherapy. (abbreviations’ definitions can potentially be omitted).

**Table 2:** Demographic and relevant clinical information.

Baseline Characteristics	Number of Patients, *n* = 47 (% of Total)
Sex (M, F)	28, 19 (59.6%, 40.4%)
Mean age at enrollment (Mean ±SD)	62 ± 11
Mean age at diagnosis (Mean ± SD)	55.77 ± 10.23
**Primary tumor**	
Midgut NET(MNET)	20 (42.5%)
Pancreatic NET(PNET)	13 (27.7%)
Other	14 (29.8%)
Lung NET	3
Rectal NET	2
Ovarian NET	1
Pheochromocytoma	1
Renal NET	1
Eustachian tube NET	1
Thymic NET	1
Ampullary NET	1
Unknown primary	3
**Grade**	
Grade 1 (Ki-67 ≤ 2)	15 (31.9%)
Grade 2 (Ki-67 3–20)	32 (68.1%)
**Chromogranin A**	
≤110	14 (30%)
>110	33 (70%)
**ECOG performance**	
0	23 (48.9%)
1	24 (51.1%)
2	0 (0%)
**Previous treatments**	
Surgery	38 (80.9%)
Non-surgical treatments:	
chemotherapy	30 (63.8%)
radiation	12 (25.3%)
liver targeted therapy	8 (17%)
Total	14 (29.8%)
**Site of metastases**	
Liver	37 (78.7%)
Lymph node	24 (51%)
Peritoneum or mesentery	9 (19.1%)
Bone	15 (31.9%)
Lung	4 (8.5%)
Other	7 (14.9%)
**Baseline NET status**	
Progressive NET	46 (97.9%)
Inoperable recurrence	1 (2.2%)

NET: neuroendocrine tumor. SD: Standard deviation.

**Table 3:** Response rate based on RECIST 1.1 and Choi criteria.

Disease Status	RECIST 1.1, *n* = 47(%)	Choi Criteria, *n* = 47(%)
Complete Response (CR)	2 (4.3%)	2 (4.3%)
Partial Response (PR)	13 (27.7%)	19 (40.4%)
Stable Disease (SD)	25 (53.1%)	18 (38.3%)
Progressive Disease (PD)	7 (14.9%)	8 (17%)

**Table 4:** Response rate based on primary site of tumor.

Primary Site of Tumor	RECIST 1.1				
	CR	PR	SD	PD	Total
**Midgut NET**	0	1 (5%)	16 (80%)	3 (15%)	20
**PNET**	0	9 (69.2%)	1 (7.7%)	3 (23.1%)	13
**Others**	2 (14.3%)	3 (21.4%)	8 (57.2%)	1 (7.1%)	14
**Total**	2 (4.3%)	13(27.7%)	25 (53.1%)	7 (14.9%)	47

CR: complete response. PR: partial response. PD: progressive disease. SD: stable disease.
